# Comprehensive behavioral analyses of mice with a glycine receptor alpha 4 deficiency

**DOI:** 10.1186/s13041-023-01033-x

**Published:** 2023-05-22

**Authors:** Mohamed Darwish, Satoko Hattori, Hirofumi Nishizono, Tsuyoshi Miyakawa, Nozomu Yachie, Keizo Takao

**Affiliations:** 1 Department of Behavioral Physiology, Graduate School of Innovative Life Science, University of Toyama, Toyama, Japan; 2 Department of Biochemistry, Faculty of Pharmacy, Cairo University, Cairo, Egypt; 3 Synthetic Biology Division, Research Center for Advanced Science and Technology, The University of Tokyo, Tokyo, Japan; 4 Division of Systems Medical Science, Center for Comprehensive Medical Science, Fujita Health University, Aichi Toyoake, Japan; 5 Medical Research Institute, Kanazawa Medical University, Kahoku, Ishikawa Japan; 6 School of Biomedical Engineering, The University of British Columbia, Vancouver, Canada; 7 Department of Behavioral Physiology, Faculty of Medicine, University of Toyama, Toyama, Japan; 8 Research Center for Idling Brain Science, University of Toyama, Toyama, Japan

**Keywords:** Glycine receptor alpha 4 subunit, Comprehensive behavioral analysis, Startle disease, Social behavior, Anxiety, Mutant mice, Brain, Spatiotemporal expression

## Abstract

Glycine receptors (GlyRs) are ligand-gated chloride channels comprising alpha (α1–4) and β subunits. The GlyR subunits play major roles in the mammalian central nervous system, ranging from regulating simple sensory information to modulating higher-order brain function. Unlike the other GlyR subunits, GlyR α4 receives relatively little attention because the human ortholog lacks a transmembrane domain and is thus considered a pseudogene. A recent genetic study reported that the GLRA4 pseudogene locus on the X chromosome is potentially involved in cognitive impairment, motor delay and craniofacial anomalies in humans. The physiologic roles of GlyR α4 in mammal behavior and its involvement in disease, however, are not known. Here we examined the temporal and spatial expression profile of GlyR α4 in the mouse brain and subjected *Glra4* mutant mice to a comprehensive behavioral analysis to elucidate the role of GlyR α4 in behavior. The GlyR α4 subunit was mainly enriched in the hindbrain and midbrain, and had relatively lower expression in the thalamus, cerebellum, hypothalamus, and olfactory bulb. In addition, expression of the GlyR α4 subunit gradually increased during brain development. *Glra4* mutant mice exhibited a decreased amplitude and delayed onset of the startle response compared with wild-type littermates, and increased social interaction in the home cage during the dark period. *Glra4* mutants also had a low percentage of entries into open arms in the elevated plus-maze test. Although mice with GlyR α4 deficiency did not show motor and learning abnormalities reported to be associated in human genomics studies, they exhibited behavioral changes in startle response and social and anxiety-like behavior. Our data clarify the spatiotemporal expression pattern of the GlyR α4 subunit and suggest that glycinergic signaling modulates social, startle, and anxiety-like behaviors in mice.

**Supplementary Information:**

The online version contains supplementary material available at 10.1186/s13041-023-01033-x.

## Introduction

Glycine is a major inhibitory neurotransmitter in the central nervous system (CNS) that controls a variety of motor and sensory functions. Glycine receptors (GlyRs) are ligand-gated chloride channels that mediate inhibitory neurotransmission in the spinal cord and other regions of the CNS. GlyRs belong to the pentameric Cys-loop receptor family and comprise different alpha and beta subunits that combine to form ion channels [1]. Four alpha subunits (α1–α4) and 1 beta subunit have been identified to assemble in a stoichiometry of 4 alpha and 1 beta subunits [2, 3]. Each subunit comprises a large extracellular N‐terminal domain, 4 transmembrane segments (TM1–TM4), a long intracellular loop connecting M3 and M4, and a short extracellular C‐terminal region [4]. The alpha subunits have more than 80% overall sequence identity and interact with synaptic membranes through the beta subunit.

GlyR subunit expression is regionally and developmentally regulated in the CNS, and the subunits show distinct regulation during brain development [5]. After birth, α1, α3, and β subunit transcripts accumulate while α2 transcripts decrease [5]. In contrast to other GlyR subunits, the α4 subunit mRNA has not been identified in localization and temporal expression studies, probably due to its low abundance in the mammalian CNS [6]. Consistent with the distinct developmental and spatial distribution pattern of GlyR subunits, the subunits display specific functional differences in the mammalian CNS and are involved in distinct neurologic/neuropsychiatric disorders [7–10]. The function of GlyR subunits other than the α4 subunit in the mammalian CNS and their roles in behavior are well-studied. The α4 subunit has received relatively little attention, however, likely because the human ortholog, *GLRA4*, is considered to be a pseudogene containing a stop codon in exon 9 in TM4 [6, 11–13].

We recently showed that GlyR α4 is expressed in mouse embryos and facilitates preimplantation embryonic development [14]. Moreover, GlyR α4 was shown to be expressed in cholinergic amacrine cells of mouse retina and cooperates with GlyR α2 to mediate glycinergic inhibitory postsynaptic currents activity in the retina [15, 16]. Although the role of the GlyR α4 subunit has not been studied in the mammalian CNS, several pieces of information suggest that it has a role in the brain and in mammal behavior as well. First, human *GLRA4* is potentially involved in intellectual disability and behavioral abnormalities [17], and disease association studies indicate that it may be associated with several neurologic/neuropsychiatric diseases, including startle disease and autism spectrum disorder [18]. Second, a recent report demonstrated that GlyR α4 contributes to touch-evoked escape behaviors in zebrafish [13]. In the present study, we aimed to characterize the spatial and temporal expression profile of GlyR α4 in the brain and reveal its physiologic roles in mammal behavior.

Our findings revealed that the GlyR α4 subunit has the highest expression in the brainstem, and the expression gradually increases during brain development. To clarify the physiologic roles of GlyR α4, we subjected *Glra4* mutant mice to a comprehensive behavioral test battery. The mutant mice exhibited a wide variety of behavioral phenotypes such as increased anxiety-like behavior, an impaired startle response, and enhanced social behavior compared with the wild-type (WT) controls.

## Methods

### Animals

We used *Glra4* mutant mice, harbor an 11-bp deletion in exon 4, prepared as described previously [14]. To avoid potential off-target effects, the mutant mice were backcrossed with C57BL/6J mice for at least 4 generations. Because *Glra4* is located on the X chromosome [19] and male and female mice have different doses of the gene, same-sex mice were used for all experiments. Hemizygous mutant males (*Glra4*^−/Y^) and their WT (*Glra4*^+/Y^) littermates were used for the behavioral analyses, and quantitative reverse transcription-polymerase chain reaction (RT-qPCR) and Western blotting experiments. The mice were maintained under a 12:12-h light:dark cycle at 22 ± 2 °C and relative humidity of 40% to 60% and with ad libitum access to chow (CE2, CLEA Japan, Inc.; CRF-1, Oriental Yeast Co., LTD., Tokyo, Japan) and water.

### RNA isolation and cDNA synthesis

RNA isolation and subsequent RT-qPCR reactions were performed according to the MIQE guidelines [20]. Mice were decapitated, and brains were collected intact or dissected into different regions according to experiment purpose and rapidly frozen in liquid nitrogen. Total RNA was extracted from mouse brain tissues using TRIzol (Invitrogen; Carlsbad, CA, USA) following the manufacturer’s protocol. The RNA quality and quantity were assessed on a BioSpec-nano spectrophotometer (Shimadzu, Kyoto, Japan) and the A260/A280 ratio was confirmed to be 1.9–2.2. All extracted RNA samples were treated enzymatically by DNAse I (Turbo DNAse; Ambion Inc., Austin, TX, USA) and 1 µg RNA was used to synthesize complementary DNA (cDNA) using the PrimeScriptTM 1st strand cDNA Synthesis Kit (TaKaRa Bio Inc.) according to the manufacturer's protocols. The cDNA template was diluted 1/20 for further use in RT-qPCR.

### RT-qPCR

Primers were designed using the PrimerQuest® Tool and Primer3 software. The primer pairs used in this study are shown in Additional file 1: Table S1. The optimum annealing temperature for all primers was tested using gradient PCR. Primer specificity was confirmed by the appearance of a single band on the gel and a single peak on the melting curve of the qPCR reaction. The efficiency of each set of primers was assessed by RT-qPCR on serial dilutions of cDNA from brain tissues and was confirmed to be in the range of 90% to 110%. PCR was performed in a 20 µl volume containing 2 µl template, 1 µl of 10 µM of each primer, 0.2 µl Phusion DNA Polymerase, 4 µl of 5 × Phusion HF Buffer (New England Biolabs, Ipswich, MA, USA), and 2 µl of 10 mM dNTPs with the following thermal cycler conditions: 98 °C for 30 s, 30 cycles of 98 °C for 10 s, 60 °C for 10 s, and 72 °C for 10 s, and then 72 °C for 5 min for the final extension. Each biological sample had 3 or 4 technical replicates and the mean of the triplicates or quadruplicates were taken to be the Ct representing the biological sample. The Ct values of the housekeeping genes were used to normalize target genes and the fold change (log2) for each sample was calculated relative to the reference sample using the 2^−ΔΔCt^ method.

### Western blotting

Brain samples were collected, frozen immediately in liquid nitrogen, and stored at − 80 °C until use. The samples were homogenized in ice-cold RIPA lysis buffer (50 mM Tris HCl, pH 8, 150 mM NaCl, 0.1% Triton X100, 0.5% sodium deoxycholate, and 0.1% sodium dodecyl-sulfate) supplemented with 1 protease inhibitor tablet (cOmplete™, Mini Protease Inhibitor Cocktail; Roche, Basel, Switzerland) using a sonicator. The lysates were centrifuged at 14,000 rpm for 20 min at 4 °C and the supernatants were stored at − 30 °C until use or used immediately for protein quantification with a protein assay bicinchoninate kit (Nacalai Tesque, Inc., Kyoto, Japan). Equal amounts of lysates in each sample were mixed with 2 × Laemmli Sample Buffer (Bio-Rad Laboratories, Inc., Hercules, CA, USA) for protein denaturation at 95 °C for 5 min. To detect proteolipid protein 1 (PLP1), the denaturation step was skipped because PLP1 aggregates in boiling lysates [21]. The lysates were then subjected to sodium dodecyl-sulfate polyacrylamide gel electrophoresis and transferred to transblot turbo polyvinylidene difluoride transfer packs using the Trans-Blot Turbo System (Bio-Rad Laboratories, Inc.). The membranes were then blocked in Tris-buffered saline with 0.1% Tween (TBST) containing 5% (w/v) non-fat milk for 1 h at room temperature to block nonspecific protein binding sites. The membranes were then incubated overnight with primary antibodies diluted in blocking buffer; rabbit polyclonal anti GlyR α4 (Cat. No. orb157164, 1:3000 dilution; Biorbyt, Cambridge, UK), rabbit mAb anti-PLP1 (Cat. No. ab254363, 1:2000 dilution, Abcam, Cambridge, UK), and rabbit mAb anti-glyceraldehyde 3-phosphate dehydrogenase (GAPDH) (Cat. No. 2118S, 1:5000 dilution; Cell Signaling Technology, Danvers, MA, USA). These antibodies had high specificity for the target proteins in mice. After washing 3 times with TBST for 5 min/wash, the membranes were incubated for 1 h at room temperature with anti-rabbit IgG, horseradish peroxidase-linked antibody diluted in blocking buffer (cat. no. 7074, 1:3000 dilution; Cell Signaling Technology). Membranes were again washed 3 times in TBST for 5 min/wash. To confirm the absence of short fragments of GlyR α4 in *Glra4* mutant mice, the membrane was first incubated with anti-GlyR α4 antibody and then stripped with stripping buffer and re-probed with anti-GAPDH. Proteins bands were detected using Clarity Western ECL substrate (Bio-Rad Laboratories, Inc.), and then the blots signals were captured using a Luminescent Image Analyzer LAS-3000 (FUJIFILM, Tokyo, Japan).

### Experimental design of behavioral analysis

*Glra4*^−/Y^ mice (n = 18) and control (*Glra4*^+/Y^) mice (n = 14) were subjected to a comprehensive behavioral test battery [22]. The behavioral tests were conducted in the following order (see Table 1): general health and neurologic screening, light/dark transition, open field, elevated plus-maze, hot plate, social interaction test in a novel environment, rotarod, three-chamber social approach, startle response/prepulse inhibition, Porsolt forced swim, T-maze, Barnes circular maze, tail suspension, contextual and cued fear conditioning tests, and social interaction test in the home cage. Super hypochlorous water and 70% ethanol were used to clean each apparatus between animals to prevent bias due to olfactory cues. The interval between tests was at least 1 day. All behavioral testing was performed between 9:00 a.m. and 6:00 p.m. Raw data from the behavioral tests and information about each mouse are accessible on the public database “Mouse Phenotype Database” (http://www.mouse-phenotype.org/).

**Table 1:** Schedule and summary of the comprehensive behavioral analysis of Glra4 mutant mice

Order	Test	Age* (w)	Figures	Phenotype of *Glra4* mutant mice
1	General health and neurologic screening	13	Additional file 3: Fig. S1	NS
2	Light–dark Transition test	13	Fig. 4	NS
3	Open field test	14	Fig. 4	NS
4	Elevated plus-maze test	14	Fig. 4	**↑** anxiety-like behavior
5	Hot plate test	14	Additional file 3: Fig. S1	NS
6	Social interaction test	15	Fig. 6	NS
7	Rotarod test	15	Additional file 3: Fig. S1	NS
8	Three-chambered social interaction test	15	Fig. 6, Additional file 3: Fig. S2	NS
9	Startle response/Prepulse inhibition test	16	Fig. 5	↓ and delayed startle response
10	Porsolt Forced Swim test	16	Additional file 3: Fig. S3	NS
11	T-maze forced alternation test	17	Additional file 3: Fig. S4	NS
12	Barnes maze test	18	Additional file 3: Fig. S4	NS
13	Tail suspension test	32	Additional file 3: Fig. S3	Mild ↓depressive-like behavior
14	Contextual and cued fear conditioning	34	Additional file 3: Fig. S4	NS
15	Social interaction in home cage	38	Fig. 6	**↑** social interaction

NS: No significant differences. *Age (weeks old) of the youngest mice at the start of the test. The oldest mouse was 7 weeks older than the youngest mouse. ↑: Increase ↓: Decrease

### General health and neurologic screening

In the general health and neurologic screens, body weight, body temperature, and muscle strength were measured, and righting, whisker twitch, ear twitch, and key jangling reflexes were evaluated. In addition, the presence of whiskers or bald hair patches was recorded. Neuromuscular strength was assessed using the wire hang and grip strength tests. In the wire hang test, each mouse was placed on a wire mesh, and the latency to fall after being inverted was recorded with a 1-min cut-off time. Forelimb grip strength was assessed using a grip strength meter (O’Hara & Co., Tokyo, Japan). Mice were lifted and held by the tail so they would grasp a wire grid using their forepaws. The mice were then gently pulled backward by the tail until they released the grid. The peak force applied by the forelimbs of each mouse was recorded in Newtons (N). For each mouse, the test was repeated 3 times and the largest value was used for statistical analysis.

### Light/dark transition test

The light/dark transition test was performed as previously described [23]. The apparatus consisted of a cage (21 × 41.5 × 25 cm) divided into 2 sections of equal size by a partition with a door (O’Hara & Co.). One chamber was dark (< 5 lx), while the other was brightly illuminated (~ 390 lx). Mice were placed into the dark chamber; the door was opened after 3 s and the mice were allowed to move freely between the chambers for 10 min. The following parameters were recorded automatically using ImageLD software (see section, “Image analysis”): total number of transitions between chambers, time spent in each chamber (s), latency to first enter the light chamber (s), and distance traveled in each chamber (cm).

### Open field test

The apparatus comprised a square cage (42 × 42 × 31 cm; Accuscan Instruments, Columbus, OH, USA) illuminated at 100 lx. Each mouse was placed in the corner of the open field apparatus and allowed to move freely while being recorded for 120 min. The following parameters were measured: total distance traveled (cm), vertical activity (rearing measured by counting the number of photobeam interruptions), time spent in the center area (s) (20 × 20 cm), and beam-break counts for stereotyped behaviors.

### Elevated plus-maze test

The elevated plus-maze test was performed as previously described [24]. The apparatus consisted of 2 arms (25 × 5 cm) with 3 mm-high ledges along the sides and distal end (open arms) and 2 enclosed arms (25 × 5 cm) with 15-cm high transparent walls along the sides and distal end (closed arms) (O'Hara & Co.). Arms of the same type were arranged opposite each other, and the arms and the central square (5 × 5 cm) were made of white plastic plates. The maze was elevated 50 cm above the floor. Each mouse was placed in the central square of the maze, facing a closed arm, and was recorded for 10 min. The illumination level at the center of the maze was 100 lx. The following parameters were calculated automatically using ImageEP software (see section, “Image analysis”): percentage of entries into the open arms, time spent in the open arms (s), total number of entries, and total distance traveled (cm).

### Rotarod test

The rotarod test was performed on rotating drums (3 cm diameter) using an accelerating rotarod (UGO Basile Accelerating Rotarod, Varese, Italy). The speed of the rotarod accelerated from 4 to 40 rpm over a 5-min period. The time each animal was able to maintain its balance on the rod was measured (s).

### Startle response/prepulse inhibition test

The acoustic startle response/prepulse inhibition test was measured using a startle reflex measurement system (O’Hara & Co.) as previously described [25]. Each mouse was gently placed in a Plexiglas cylinder where it was left undisturbed for 10 min as a test session. White noise (40 ms) was used as the startle stimulus for all trial types and the startle response was recorded for 400 ms (measuring the response every 1 ms) starting with the onset of the startle stimulus. The background noise level in each chamber was 70 dB. The peak startle amplitude recorded during the 200-ms sampling window was used as the dependent variable. The intensity of the startle stimulus was 110 or 120 dB. The prepulse sound was presented 100 ms before the startle stimulus, and the intensity was either 74 or 78 dB. Four combinations of prepulse and startle stimuli were used (74, 110; 78,110; 74,120; and 78, 120 dB). Six trial types were used in each session (i.e., 2 types for startle stimulus-only trials, and 4 types for prepulse inhibition trials). Six sessions of the 6 trial types were presented in a pseudorandom order such that each trial type was presented once within a block. The average inter-trial interval was 15 s (range 10–20 s).

### Tail suspension test

The tail suspension test was performed in white plastic chambers (33 × 56 × 45 cm) (O’Hara & Co.) where each mouse is suspended by its tail with adhesive tape 30 cm above the floor. The behavior of the mouse was recorded with a video camera for 10 min. The immobility time was measured using ImageTS software (see section, “Image analysis”). Immobility was defined as time spent not moving that lasted more than 2 s.

### Porsolt forced swim test

The Porsolt forced swim test was performed in plastic cylinders (height 22 cm, inner diameter 11 cm) (O’Hara & Co.) were filled with super hypochlorous water (approximately 23 °C) to a height of 7.5 cm. The mice were placed in the cylinder and allowed to swim for 10 min; the immobility time and distance traveled were recorded using ImageTS software (see section, “Image analysis”). Immobility was defined as time spent not moving that lasted more than 2 s.

### Social interaction test in a novel environment

The social interaction test in a novel environment was performed as previously described [25]. Two mice of the same genotype that had never before been exposed to each other were placed in a box (40 × 40 × 30 cm) and allowed to move freely for 10 min. Several parameters, such as number of contacts, number of active contacts, contact duration (s), total distance traveled (cm), and mean duration/contact, were analyzed using ImageSI software (see section, “Image analysis”). An active contact was defined as when the 2 mice traveled together for at least 10 min.

### Three-chambered social approach test

The sociability and social novelty preference tests were performed as previously described [26, 27]. The apparatus consisted of a rectangular, 3-chamber lidded box with a video camera (O’Hara & Co.). The 3 chambers (each 20 × 40 × 46.5 cm) were arranged side-by-side, each pair separated by a dividing wall with a small square opening (5 × 3 cm) to allow the mice to freely navigate among the chambers. In this test, unfamiliar mice that had no earlier contact with the test mice were enclosed in round cages (height 11 cm, bottom diameter 9 cm, vertical bars 0.5 cm apart) that allowed nose contact but prevented aggressive behavior. An unfamiliar C57BL/6 J male mouse (stranger 1) in a round cage was placed in 1 of the side chambers, an empty round cage was placed in the same relative location in the other side chamber, and the test mouse was placed in the middle chamber and allowed to explore the entire social test box for 10 min. The time the test mouse spent in each chamber was measured to quantify sociability for the first stranger compared with the empty cage. For the next session, another unfamiliar mouse (stranger 2) was placed in the chamber that was empty during the first 10-min session. The test mouse was then placed in the middle chamber and allowed to explore for 10 min and to choose between the first, already-investigated unfamiliar mouse (stranger 1) and the novel unfamiliar mouse (stranger 2). The time spent in each chamber during the second 10-min session was measured to quantify social novelty preferences. Data acquisition and analysis were performed automatically using ImageCSI software (see section, “Image analysis”).

### Home cage social interaction test

Home cage social interaction monitoring was performed as previously described [26]. The system comprised a home cage (29 × 19 × 13 cm) and a filtered cage top containing an infrared video camera. Two mice of the same genotype, which had been housed separately, were placed together in the home cage. Their social interaction and locomotor activity were monitored for 1 week. Social interaction was measured by counting the number of particles detected in each frame: 1 particle indicates that the 2 mice are close to each other whereas 2 particles indicate that the 2 mice are apart from each other. The analysis was performed automatically using ImageHC software (see “Image analysis”).

### Barnes maze test

The Barnes circular maze test was conducted on a white circular surface, 1.0 m in diameter, with 12 holes equally spaced around the perimeter (O’Hara & Co.). A black Plexiglas escape box (17 × 13 × 7 cm) containing paper cage bedding was located under 1 of the holes. For each mouse, the location of the target was consistent but randomized across mice. The maze was rotated after each trial, and to prevent bias based on the proximal cues within the maze, the spatial location of the target was unchanged with respect to the distal visual room cues. Two trials per day were conducted over 8 training sessions for a total of 16 trials. The latency (s) to reach the target hole, the number of errors, and distance (cm) to reach the target hole were recorded using ImageBM software (see section, “Image analysis”). The day after the last training session, a probe test was conducted without the escape box for 3 min, and the time spent around each hole was recorded to test spatial memory. One month later, a second probe test was performed to assess long-term memory retention.

### Fear conditioning test

The contextual and cued fear conditioning test was performed as previously described [28]. Each mouse was placed in a transparent acrylic chamber (26 × 34 × 33 cm) with a grid floor (O’Hara & Co.) and allowed to explore freely for 2 min. A 55-dB white noise was presented for 30 s as a conditioned stimulus (CS), followed by a mild footshock (0.3 mA) during the last 2 s of the CS as an unconditioned stimulus (US). To strengthen the association, 2 more CS-US pairings were presented with a 2-min interstimulus interval to complete the conditioning session. Contextual testing and cued testing were conducted both 1 day and 1 month after the conditioning session. In the contextual testing, each mouse was placed in the same test chamber as used for the conditioning session for 5 min without any stimulus. In the cued testing, each mouse was placed in a triangular chamber (33 × 33 × 33 cm) made of white opaque Plexiglas for 6 min. In the first 3 min, no CS or US was presented, whereas in the last 3 min, the CS was presented. In the conditioning, context, and cued testing, freezing percentage and distance traveled (cm) were calculated using ImageFZ software (see section, “Image analysis”). Freezing was defined as a complete lack of movement of any part of the body for at least 2 s.

### T-maze forced alternation test

The T-maze test was performed using an automatic, modified T-maze apparatus (O’Hara & Co.) as previously described [29]. The T-maze comprised white plastic runways with 25-cm high walls partitioned off into 6 areas by sliding doors that open downward automatically. The stem of the T was the S2 area (13 × 24 cm) and the 2 other arms of the T shape were the target arms (A1 and A2, 11 × 20.5 cm each). A1 and A2 connected to the starting area S1 by connecting passages. In the forced alternation task without food, each mouse was subjected to 10 consecutive trials in a session/day for 3 days; each trial comprised a forced-choice run followed by a free-choice run. Each mouse was forced to choose 1 of the target arms of the T (forced-choice run); the door was opened after 10 s and the mouse was returned to the starting area (S1) and allowed to freely choose between both target arms (free-choice run). The percentage of correct trials in which the mice chose the arm opposite their forced-choice run was calculated. The mice were then subjected to the delayed alternation task where 3-, 30-, 60-, or 120-s delays were inserted between the forced- and free-choice runs. Data analysis and acquisition were performed automatically using ImageTM (see section, “Image analysis”).

### Image analysis

Behavioral data were collected automatically using the following applications (ImageLD, EP, SI, CSI, BM, FZ, TM, HA, and TS) based on the public domain ImageJ program, and were modified for each test by Tsuyoshi Miyakawa and his colleagues.

### Statistical analysis

For the behavioral experiments, statistical analysis was conducted using StatView (SAS Institute, Cary, NC) and data were analyzed using 2-tailed t-tests, paired t-test, one-way analysis of variance (ANOVA), or two-way repeated-measures ANOVA. In the behavioral test battery, we run a series of tests which increases type I error; thus, we consider the results should be corrected for multiple comparisons. For multiple testing in the behavioral test battery, we defined study-wide significance as the statistical significance that survived the false discovery rate (FDR) [30, 31]. Nominal significance was defined as a difference in an index between groups that achieved statistical significance (p < 0.05) but did not survive the FDR correction. All statistical analysis values for the behavioral analyses are included in Additional file 2: Table S2. Statistical analysis for RT-qPCR and Western blotting data was conducted using GraphPad Prism version 8.0.0 (GraphPad Software, Inc., San Diego, CA) and analyzed using Welch’s t or Mann–Whitney tests. Values in graphs and tables are expressed as mean ± SEM.

## Results

### Differential temporal expression patterns of mouse GlyR subunits in the brain

**Fig. 1:**
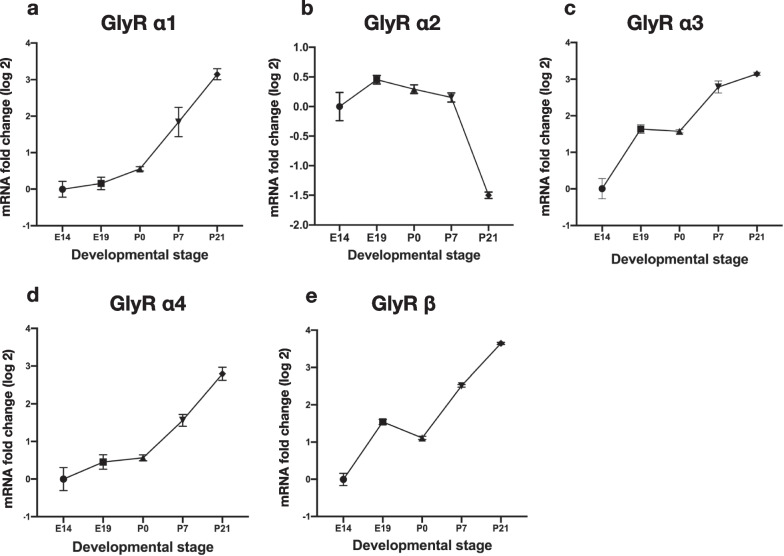
Temporal expression of GlyR subunit mRNA during mouse brain development. RT-qPCR analysis of **a** GlyR α1, **b** GlyR α2, **c** GlyR α3, **d** GlyR α4, and **e** GlyR β subunits in various developmental stages (E14, E19, P0, P7, and P21). *Rpl13a*, *Gus*, *and Ywha*z were used as controls. Data are presented as mean ± SEM of fold-change (log 2). Results are from at least 3 biologic replicates in each group, and the experiments were carried out in quadruplicate

To precisely determine the timing and sequence of changes in the expression of mouse GlyR subunits during development, we studied the temporal expression pattern of each subunit (GlyR α1–4 and β) during brain development at E14, E19, P0, P7, and P21. We detected the mRNA transcripts of all GlyR subunits at E14 (Fig. 1). GlyR α1 mRNA expression progressively increased during mouse brain development, with significant growth from P0 to P21 (Fig. 1a). In contrast to GlyR α1, GlyR α2 mRNA expression gradually decreased during brain development, specifically from E19 to P21 (Fig. 1b). These data confirm the phenomenon of a developmental switch between the α1 and α2 subunits that occurs nearly 2 weeks postnatally and has been reported in rat brain and mouse spinal cord [5, 32]. Similar to the α1 subunit, mRNA expression of the GlyR α3, α4, and β subunits gradually increased during brain development (Fig. 1c-e). Overall, the GlyR subunit transcript expression exhibited a ~ tenfold increase during development from E14 to P21, but transcript expression of the α3 and β subunits reached a fourfold increase prenatally at E19, and that of the α1 and α4 subunits reached a fourfold increase postnatally at P7. Taken together, transcript expression of all GlyR subunits increased during brain development from E14 to P21 except for α2, which decreased by ~ threefold.

### Regional distribution pattern of GlyR α4 in mouse brain

**Fig. 2:**
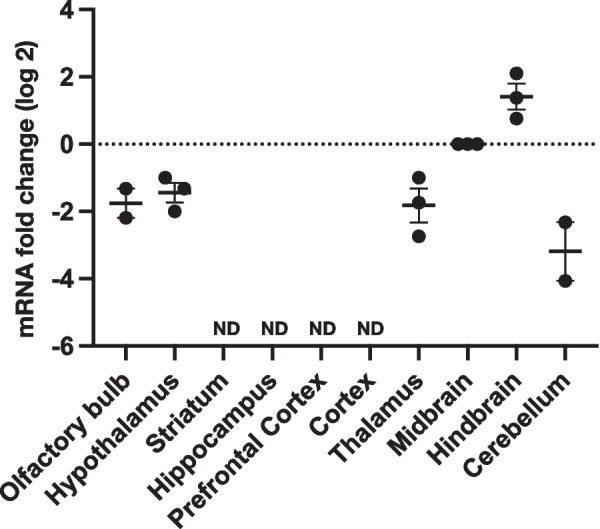
Distribution pattern of the GlyR α4 subunit in various brain regions. RT-qPCR analysis of GlyR α4 in the olfactory bulb, hypothalamus, striatum, hippocampus, prefrontal cortex, cortex, thalamus, midbrain, hindbrain, and cerebellum in mouse brain (6 weeks). Data are presented as individual values of fold-change (log 2), results are from 3 biologic replicates in each group, and the experiments were carried out in triplicate. (*Gapdh* and *Actb* used as controls). ND, not detected

To characterize the spatial distribution of GlyR α4, we dissected the mouse brain (6 weeks) into 10 regions (olfactory bulb, hypothalamus, striatum, hippocampus, prefrontal cortex, cortex, thalamus, midbrain, hindbrain, and cerebellum) and quantified the transcript expression levels using RT-qPCR. The highest expression of GlyR α4 mRNA was detected in the hindbrain and midbrain, and lower expression levels were detected in the olfactory bulb, hypothalamus, thalamus, and cerebellum. The GlyR α4 subunit mRNA was not detected in the striatum, hippocampus, prefrontal cortex, or cortex (Fig. 2). These findings together indicate that GlyR α4 transcript expression predominates in the brainstem, consistent with the general localization pattern of GlyRs.

### Characterization of *Glra4* mutant mice

**Fig. 3:**
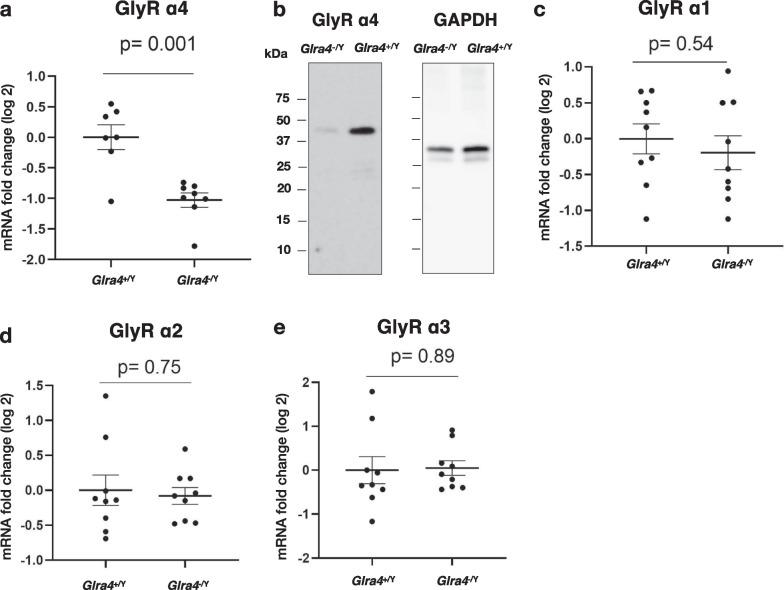
Characterization of *Glra4*^−/Y^ mice. **a** RT-qPCR analysis of mRNA derived from exons 3 and 4 of GlyRα 4 gene in the hindbrain of WT and *Glra4*^−/Y^ mice. Data are presented as mean ± SEM of fold-change (log 2), n = 7 for WT and n = 8 for *Glra4*^−/Y^ mice, and the experiments were carried out in triplicate. The p-value was determined by Welch’s t-test. **b** Whole western blot image of brain lysates from *Glra4*^−/Y^ and *Glra4*^+/Y^ mice against GlyR α4 (left) and GAPDH after membrane stripping (right). **c**–**e** RT-qPCR analysis of **c** GlyR α1, **d** GlyR α2, and **e** GlyR α3 transcripts in *Glra4*^+/Y^ and *Glra4*^−/Y^ mouse hindbrain. n = 9 *Glra4*^+/Y^, n = 8 *Glra4*^−/Y^. Data are presented as mean ± SEM of fold-change (log 2), and the experiments were carried out in triplicate. The p values were determined by Welch’s t-test

We used *Glra4*^−/Y^ mice having an 11-bp deletion in exon 4 that we previously generated in our lab [14] to explore the physiologic roles of GlyR α4. We performed RT-qPCR to quantify the GlyR α4 transcript levels in *Glra4*^−/Y^ mice: the abundance of GlyR α4 transcripts was significantly decreased in the mutant mice compared with controls (Fig. 3a). Consistently, the Western blot assay revealed that GlyR α4 protein expression was also reduced in *Glra4*^−/Y^ mice (Fig. 3b).

As the GlyR α4 subunit has other paralogs (α1, α2, and α3), we checked whether the expression of any other subunits changed to compensate for the GlyR α4 deficiency. RT-qPCR revealed no significant differences in the GlyR α1, α2, or α3 subunit transcript abundance in the *Glra4*^−/Y^ mouse hindbrain compared with the WT littermates (Fig. 3c–e). These data do not support the existence of compensatory regulation of GlyR α subunits in response to GlyR α4 depletion.

### No change in physical characteristics, muscular strength, motor function, or nociception in *Glra4* mutant mice

The *Glra4*^−/Y^ mice and their *Glra4*^+/Y^ littermates were subjected to a battery of behavioral tests to screen for behavioral phenotypes caused by GlyR α4 mutation and elucidate the physiologic roles of GlyR α4. We first checked the general health, neurologic and motor functions, and nociception. The appearance of the fur and whiskers, body weight (Additional file 3: Fig. S1a), and body temperature (Additional file 3: Fig. S1b) did not differ significantly between the *Gla4*^*−/Y*^ mice and the controls. Responses to key jangling, whisker twitch, ear twitch, and righting reflex were also similar across genotypes. In addition, the wire hang (Additional file 3: Fig. S1c), grip strength (Additional file 3: Fig. S1d), rotarod (Additional file 3: Fig. S1e), and hot plate (Additional file 3: Fig. S1f) tests revealed no significant differences between genotypes. These results suggest that *Glra4* deficiency does not affect general health, nociception, or neurologic and motor functions.

### Increased anxiety-like behavior in *Glra4* mutant mice

**Fig. 4:**
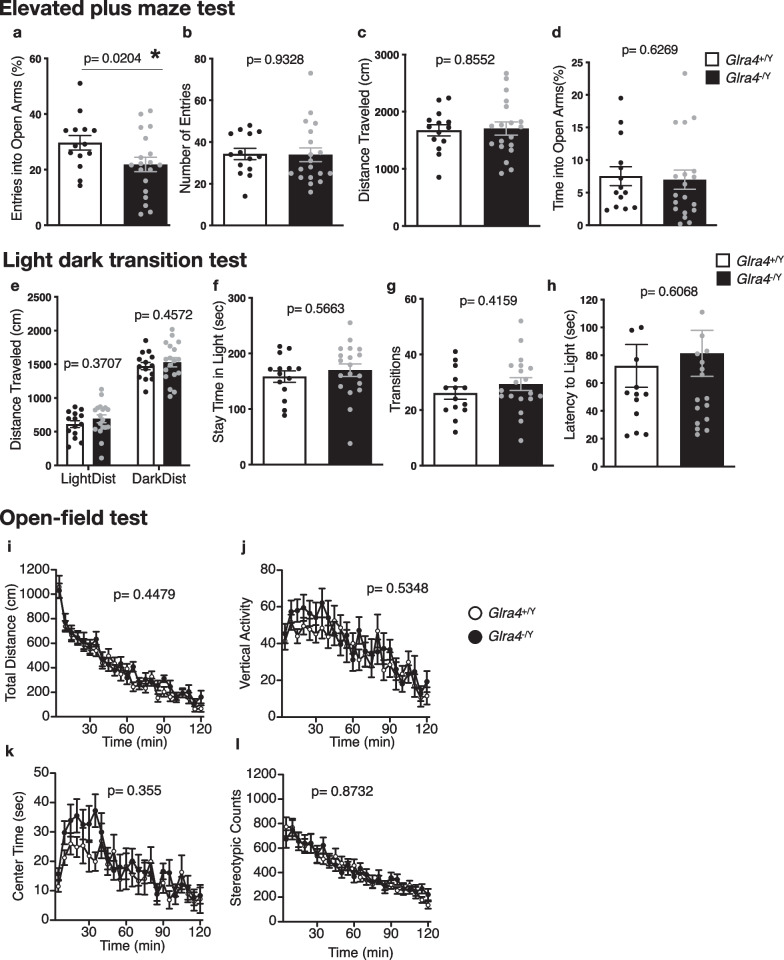
Anxiety-like behavior of *Glra4*^−/Y^ mice. **a**–**d** Elevated plus-maze test: **a** Percentage of entries into open arms, **b** Number of arm entries, **c** Total distance traveled, **d** Percentage of time spent on open arms. **e**–**h** Light/dark transition test: **e** Total distance traveled, **f** Time spent in the light compartment, **g** Light/dark transition number, **h** and Latency to enter the light compartment. **i**–**l** Open field test: **i** Total distance traveled, **j** Vertical activity count, **k** Time spent in the center area, **l** Stereotypic behavior count. n = 14 *Glra4*^+/Y^, n = 18 *Glra4*^−/Y^. Data are presented as mean ± SEM. The p values indicate the genotype effects in one-way ANOVA (**a**–**h**), and two-way repeated-measures ANOVA (**i**–**l**). Asterisk indicates nominal significance

Anxiety-like behavior was assessed using the elevated plus-maze, light–dark transition, and open field tests. In the elevated plus-maze test, the percentage of entries into the open arms was significantly decreased in *Glra4*^−/Y^ mice compared with their WT littermates (Fig. 4a). The total number of entries into arms, distance traveled, and percentage of time spent in the open arms did not significantly differ between genotypes (Fig. 4b–d). In the light–dark transition test, distance traveled, time spent in the light chamber, number of transitions, and latency to light (Fig. 4e–h) did not differ significantly between genotypes. In the open field test, total distance traveled, vertical activity, time spent in the center, and stereotypic counts (Fig. 4i–l) did not differ significantly between genotypes. These data suggest that GlyR α4 may be involved in anxiety-like behavior related to elevated spaces.

### Decreased and delayed acoustic startle response in *Glra4* mutant mice

**Fig. 5:**
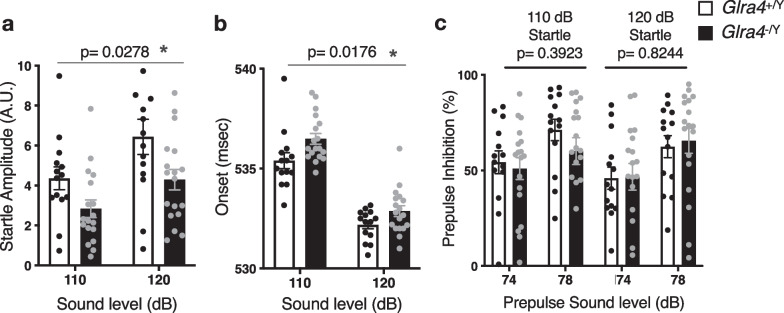
Acoustic startle response and prepulse inhibition in *Glra4*^−/Y^ mice. **a** Startle response to 110 dB and 120 dB acoustic stimuli. **b** Startle onset to 110-dB and 120-dB acoustic stimuli, **c** Prepulse inhibition of the 110-dB and 120-dB startle response with 74-dB and 78-dB prepulse sounds. n = 14 *Glra4*^+/Y^, n = 18 *Glra4*^−/Y^. Data are presented as mean ± SEM. The p values indicate the genotype effects in two-way repeated-measures ANOVA. Asterisks indicate nominal significance

GlyRs are involved in modulating the startle response in humans and rodents [1]. To assess the role of the GlyR α4 subunit in the startle reflex, we subjected the mice to the acoustic startle response and prepulse inhibition tests. The *Glra4*^−/Y^ mice displayed a significantly lower acoustic startle response (Fig. 5a). They also manifested a delayed onset of the startle response compared with their WT littermates (Fig. 5b). Prepulse inhibition did not differ significantly between genotypes (Fig. 5c). These results suggest that *Glra4* deficiency attenuates and delays the startle reflex in mice.

### Increased social interaction in *Glra4* mutant mice

**Fig. 6:**
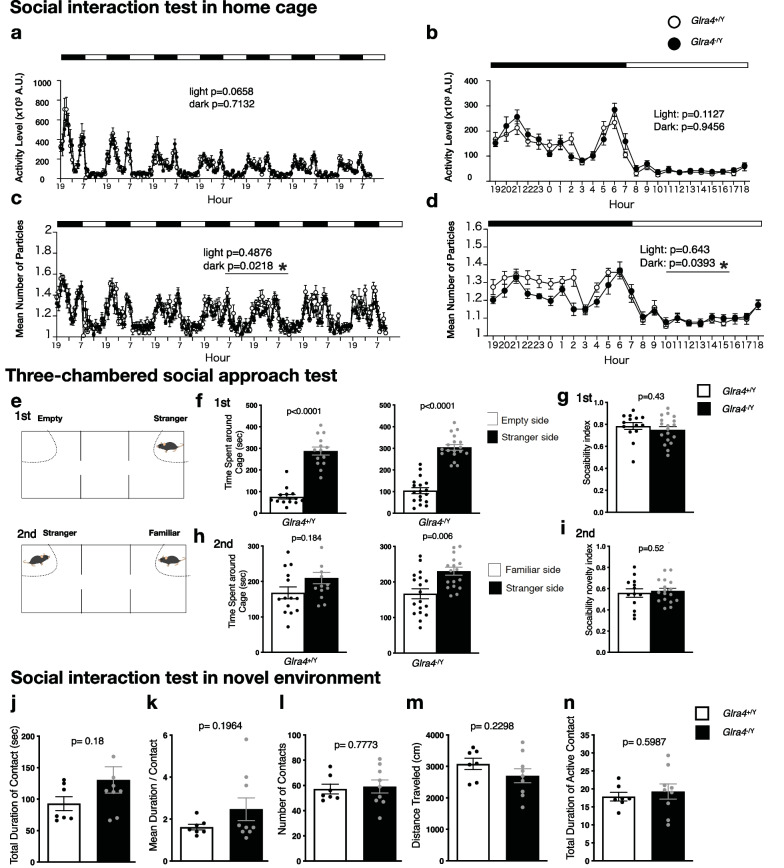
Social behavior in *Glra4*^−/Y^ mice. **a**–**d** Social interaction test in a home cage. Light and dark periods are represented by the white and black bars. **a** The total activity level through a 7-day experiment, **b** Mean total activity during 3 days from the 7-day experiment represented in **a**, **c** Mean number of particles through the 7-day experiment, **d** Mean number of particles during 3 days from the 7-day experiment represented in **c**. A particle number of 1 indicates that the 2 mice are close to each other, whereas a particle number of 2 indicates that they are apart from each other. A lower number of particles represents higher social interaction. **a**–**d** n = 7 *Glra4*^+/Y^, n = 9 *Glra4*^−/Y^. **e**–**i** The three-chambered social approach test. **e** Schematic diagram describing the test. In the first session, 1 cage is empty and the other cage contains a stranger mouse. In the second session, 1 cage contains the familiar mouse (the stranger in the first session) and the other contains a novel stranger mouse. **f** Time spent around either the empty cage or stranger mouse cage in the first session, and **h** either a familiar mouse cage or stranger mouse cage in the second session. **g**, **i** Sociability index and social novelty index calculated as the ratio of time spent around the stranger cage to that spent in all cages. **j**–**n** Social interaction test in a novel environment. **j** Total duration of contact, **k** Mean duration/contact, **l** Number of contacts, **m** Total distance traveled, **n** Total duration of active contact. **e**–**n** n = 14 *Glra4*^+/Y^, n = 18 *Glra4*^−/Y^. Data are presented as mean ± SEM. The p values indicate the genotype effects in a paired t-test (**f**, **h**), one-way ANOVA (**g**–**n**), and two-way repeated-measures ANOVA (**a**–**d**). Asterisks indicate nominal significance

To evaluate the roles of GlyR α4 in social behavior, we exposed *Glra4*^−/Y^ mice and WT littermates to 3 behavioral tests; the social interaction test in the home cage, the three-chambered social approach test, and the social interaction test in a novel environment. In the social interaction test in the home cage, 2 same-genotype mice were placed in the same cage for 1 week and their social behavior and activity were recorded. The social interaction was estimated by the mean number of particles visualized, with a lower number of particles indicating higher social interaction. The *Glra4*^−/Y^ mice showed higher social interaction than the WT mice during the dark period when mice were the most active, whereas the social interaction was comparable between *Glra4*^−/Y^ mice and WT mice during the light period (Fig. 6c, d). These data suggest that *Glra4* mutant mice had increased social interaction during their active period.

The three‐chambered social approach test was used to assess sociability and preference for social novelty. Sociability was assessed based on the preference for a cage with a stranger mouse compared with an empty cage (Fig. 6e, 1st), whereas social novelty was assessed based on preference for a cage with a stranger mouse over a cage with a familiar mouse (Fig. 6e, 2nd). In the sociability test, both control and mutant mice spent significantly more time around the cage of the stranger mouse than the empty cage, with a comparable sociability index between genotypes (Fig. 6f, g). In the social novelty test, the WT and mutant mice exhibited different preferences: in WT mice, the time spent around the cage with the stranger was not significantly different than the time spent around the cage of the familiar mouse (Fig. 6h). In contrast, the mutants spent significantly more time around the cage with the stranger mouse compared with the cage of the familiar mouse (Fig. 6h). The social novelty preference index, however, did not differ significantly between genotypes (Fig. 6i). The smaller number of mice in the control group may explain why the difference in the spent time around the stranger cage vs the familiar cage did not reach the level of significance.

In the social interaction test in a novel environment, 2 mice of the same genotype from distinct cages were placed in a novel environment and were allowed to interact with each other for 10 min. The total duration of contact, mean duration per contact, number of contacts, and total duration of active contact did not significantly differ between genotypes (Fig. 6j–l, n). In the three social behavioral tests, general activity levels including total distance traveled and average locomotion speed, did not differ significantly between genotypes (Additional file 3: Fig. S2, Fig. 6a, b, m). Taken together, these data suggest that the GlyR α4 deficiency increased social interaction but did not affect locomotor activity in mice.

### Comparable depression-related behavior and memory function in *Glra4* mutant mice and controls

In contrast to other GlyR α subunits, the GlyR α4 is enriched in myelinating oligodendrocytes [33]. Since oligodendrocytes have been reported to participate in the pathogenesis of depression and mood regulation [34], we checked whether the deficiency of GlyR α4 affects depression-related behavior in mice. We exposed the mice to the tail suspension test and Porsolt forced swim test to examine depression-related behavior in *Glra4* mutant mice. In the tail suspension test, *Glra4*^−/Y^ mice showed a mild decrease in the percentage of immobility time compared with WT mice, but the difference was not significant (Additional file 3: Fig. S3c). In the Porsolt forced swim test, there were no significant differences in the immobility percentage and distance traveled between *Glra4*^−/Y^ mice and their WT littermates (Additional file 3: Fig. S3a, b). Taken together, these data suggest that GlyR α4 is not involved in depression-related behavior in mice.

To assess learning and memory in *Glra4*^−/Y^ mice, we performed contextual and cued fear conditioning, Barnes maze, and T-maze forced alternation tests. In the 3 paradigms, there were no significant differences in the performance of *Glra4*^−/Y^ mice and their WT littermates (Additional file 3: Fig. S4). Hence, fear, and spatial and working memories were not disrupted in *Glra4* mutant mice.

### Effect of GlyR α4 mutation on transcript and protein levels of major myelin-related genes

**Fig. 7:**
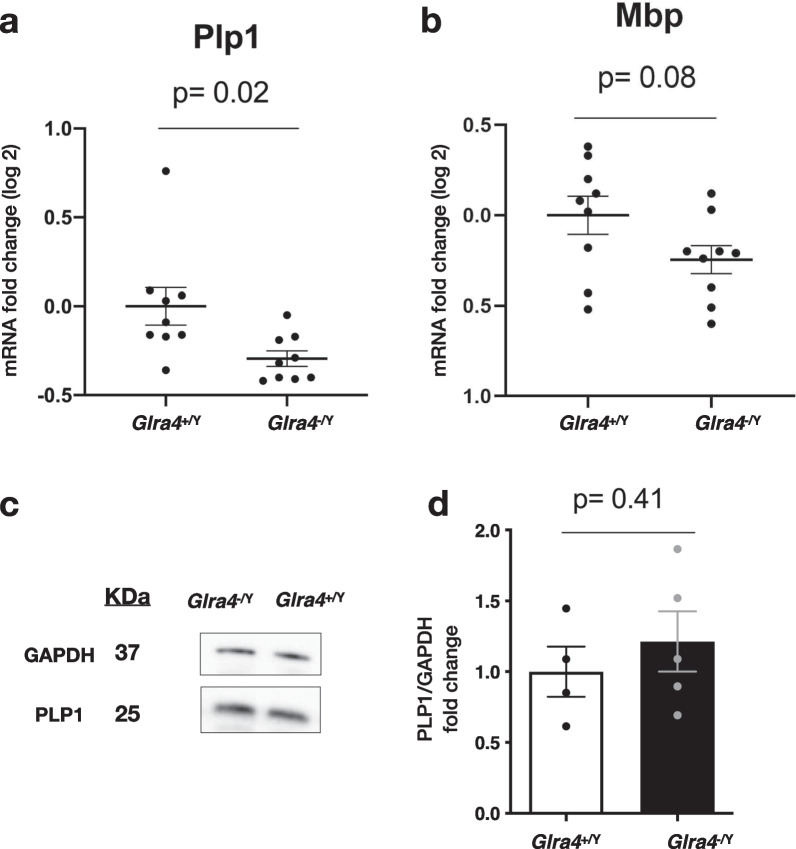
Myelin-related genes and proteins in *Glra4*^−/Y^ mice. RT-qPCR analysis of **a** *Plp*1, **b** *Mbp* transcripts in *Glra4*^+/Y^ and *Glra4*^−/Y^ mouse hindbrain. n = 9 in each group. Data are presented as mean ± SEM of fold-change (log 2) and the experiments were carried out in triplicate. Western blot analysis **c** and quantification **d** of PLP1 protein in hindbrain lysates from WT and mutant mice. GAPDH was used as a reference control. The graph in d shows densitometric quantification of Western blot bands. n = 4 *Glra4*^+/Y^, n = 5 *Glra4*^−/Y^. Data are presented as mean ± SEM. The p values were determined by Welch’s t-test (**a**, **b**) and the Mann-Whitney test (**d**)

*Glra4* mutant mice exhibited reduced and delayed startle responses. It has been reported that hypomyelination can delay the latency to perform startle responses [35]. Additionally, GlyR α4 is mainly enriched in myelinating oligodendrocytes [33]; therefore, we hypothesized that the GlyR α4 mutation would affect myelination. First, we evaluated the expression levels of major myelin-related genes such as proteolipid protein1 (*Plp1*) and myelin basic protein (*Mbp*). In the hindbrain of *Glra4*^−/Y^ mice, the mRNA transcript levels of *Plp1* and *Mbp* were either significantly reduced or had a nonsignificant tendency to be reduced, respectively, compared with WT controls (Fig. 7a, b). We then checked the myelination status by quantifying the PLP1 protein levels in the hindbrain using Western blotting. PLP1 protein levels did not differ significantly between *Glra4*^−/Y^ mice and WT littermates (Fig. 7c, d). These results suggest that the myelination levels did not differ in the *Glra4* mutant mice.

## Discussion

In the present study, we examined the spatial and temporal gene expression pattern of the previously uncharacterized GlyR α4 subunit throughout the brain using RT-qPCR and investigated the influence of GlyR α4 mutation on mouse behavior. GlyR α4 had the highest expression levels in the hindbrain and midbrain, and lower expression levels in the olfactory bulb, thalamus, hypothalamus, and cerebellum. In contrast, GlyR α4 expression was not detected in higher brain regions such as the cortex, striatum, and hippocampus. This expression pattern is consistent with the distribution profile revealed by anatomic localization of GlyR using autoradiographic labeling [1, 36]. Consistently, a gene trapping line revealed that the GlyR α4 subunit is enriched in the brainstem and spinal cord interneurons in zebrafish [13].

We characterized the genetic mutation in *Glra4* mutant mice and assessed its impact on transcript and protein levels because distinct genetic mutations lead to profound phenotypic differences. GlyR α4 transcript levels were significantly decreased in mutant mice compared with their WT littermates, probably due to the effect of nonsense-mediated mRNA decay, which is a posttranscriptional quality control mechanism [37]. Sequencing of the mutant transcript showed that the 11-bp deletion was inherited to the mRNA, which led us to expect that the induced frameshift mutation would produce a truncated protein. Unexpectedly, the size of the expressed GlyR α4 protein in the mutant mice was equivalent to that in WT mice. There were no other fragments detected at a lower size and we do not expect the absence of shorter fragments to be due to epitope binding because the antibody is supposed to bind to the N-terminus end of the GlyR α4 (Fig. 3b). The expression of intact GlyR α4 in the mutant mice may be due to illegitimate translation from out-of-frame alleles [38] where ribosomes reinitiate the translation at downstream AUG or non-AUG start codons [39, 40]. Although the size of the GlyR α4 protein was intact, the band intensity was decreased compared with that of the WT mice, consistent with the mRNA expression levels. In addition to the decreased expression of GlyR α4 protein in the mutant mice, its functionality remains unknown, but at the least the *Glra4*^*−/Y*^ mice can be considered a knockdown mouse model. Although there are still possibilities of protein expression or turnover changes in other GlyR α subunits, we failed to detect compensation among the other subunits to adapt to the mutation in GlyR α4 at the transcript level. This is probably because the *Glra4* mutant mice were a knockdown model rather than a null knockout mouse model and compensatory networks are only activated to buffer against deleterious mutations due to gene knockout, not knockdown [41]. Because the mutation type affects mouse behaviors, we do not exclude the possibility that different behavioral phenotypes would be observed in *Glra4* complete null knockout mice.

A recent study suggested that zebrafish GlyR α4a is involved in the escape and startle behaviors. Mutation or knockdown of GlyR α4a induces aberrant tactile startle and escape responses in zebrafish [13]. These findings are consistent with the present study in which mice with a GlyR α4 deficiency had a reduced and delayed startle response to an acoustic stimulus. The neural circuit involved in the acoustic startle response is located in the lower brainstem where the caudal pontine reticular nucleus receives input from cochlear neurons and transmits it toward output motor neurons [42]. The enrichment of GlyR α4 in the brainstem may explain its involvement in the startle response. Taken together, GlyR α4 may be involved in both tactile and acoustic startle responses and this role may be consistent across species. GlyRs play important roles in the pathophysiology of startle diseases: mutations in the GlyR α1 and β subtypes are involved in the exaggerated startle response in humans and mice [43–45]. Homozygous spasmodic mice harboring a mutation in *Glra1* displayed abnormally enhanced startle responses in response to acoustic stimuli [46]. By comparing the exaggerated startle response of *Glra1* mutant mice to the decreased and delayed response of *Glra4* mutant mice, we expect that mutations in the GlyR α4 and GlyR α1 genes induce distinct startle phenotypes in mice. Therefore, we do not exclude the possibility that whether the 2 GlyR subunits play opposing roles in modulating startle behavior.

Previous reports linked glycinergic transmission with agoraphobia and anxiety disorders in humans [47, 48]. Consistently, *Glrb* mutant mice exhibited increased anxiety-like behavior in the open field test, and spasmodic mice harboring a single mutation in GlyRα1 exhibited fear-related behavior in the startle paradigm [32, 47, 48]. Consistent with these reports, we show that *Glra4*-deficient mice manifested anxiety-like behavior by exhibiting a lower percentage of entries into the open arms of the elevated plus-maze test compared with controls. In contrast, they showed comparable anxiety-like behaviors in the open-field and light–dark transition tests. Although the 3 tests are used for assessing anxiety in mice, the results are not always consistent because they assess different aspects of anxiety-like behavior [49–52]. Among them, anxiety-like behavior in an elevated space was increased in GlyR α4-deficient mice.

Human *GLRA4* is considered a pseudogene because it contains a stop codon at exon 9 and lacks TM4 and is therefore likely unfunctional [13, 17]. However, there are discrepant reports about *GLRA4* implication in Pelizaeus Merzbacher Disease (PMD) [17, 53]. PMD is an intractable neurodevelopmental disease that develops due to abnormalities in myelination in the CNS and patients suffer from a constellation of motor dysfunction, impaired cognitive abilities, and hypotonia, and generally die in infancy [54]. A recent report described a young female patient suffering from cognitive impairment and motor delay with 110-kb microdeletions at Xq22.2 that encompassed 3 adjacent genes, including *GLRA4*. Among the 3 genes, only the *GLRA4* transcripts were decreased in the patient compared with her healthy mother. The authors attributed her behavior, which overlaps with PMD, to the loss of a single *GLRA4* allele [17]. In contrast, another case report ruled out the possibility that *GLRA*4 is a causative factor in a patient displaying a subset of PMD symptoms despite *GLRA4* interruption in the inverted X chromosome [53]. In our study, the cognitive and motor functions were intact in *Glra4* mutant mice compared with the WT littermates. One of the phenotypes of PMD patients is a prolonged latency in the blink reflex, a component of the startle response [55]. In the present study, the *Glra4* mutant mice manifested delayed and decreased acoustic startle response. The circuits that mediate the blink reflex and startle reaction share some commonalities through basal ganglia modulation of brainstem interneurons [56, 57]. In addition, the prolonged/delayed latency in these behaviors might occur due to demyelination or slowed conduction in the brainstem [35, 55]. The enrichment of GlyR α4 in myelinating oligodendrocytes led us to check whether myelination is affected in *Glra4* mutant mice. Although the *Plp*1 transcript expression level was significantly decreased in the mutant mice, the PLP1 protein levels did not significantly change between genotypes. The failure to detect differences in protein expression, however, does not exclude myelination abnormalities, such as changes in myelin turnover, in *Glra4* mutant mice. Further electrophysiologic experiments are required to address whether conductance or signaling is altered in *Glra4* mutant mice.

The *Glra4* mutant mice manifested enhanced social interaction in the home cage; the mutant mice spent significantly more time staying together compared to WT controls during the dark period, the period when they were active. In contrast, the social behavior did not significantly change in the one-cage novel environment nor in the three-chambered social approach test. The home cage social interaction test is a more naturalistic assay that allows continuous monitoring of mice social behavior under familiar conditions for 1 week which can capture the behavior that may not be observable with tests that last for a short time such as one-cage novel environment and the three-chamber social approach tests. This difference in the results of the social interaction tests might be due to the different conditions and methods used to asses social behavior in these assays. Therefore, we consider that *Glra4* mutant mice exhibited enhanced social behavior with familiar mice in a familiar environment resembling the real social interaction in the home cage while they did not demonstrate social behavior changes with unfamiliar mice. Consistent with this finding, a previous study showed an association between glycinergic signaling and social behavior and reported that strychnine, an antagonist for glycine receptors, improved social behavior in humans [58]. There is also a possibility that the changed social behavior in *Glra4* mutant mice during the nocturnal period is due to an alteration in circadian behavior. Glycinergic transmission is known to contribute to the synchronization of the circadian behavior and network [59, 60] and prolonged recording in the home cage social interaction test allows to analyze the interplay of circadian rhythm and social behavior and investigate the behavioral changes in a circadian-dependent manner. Therefore, it is conceivable that the mutations in glycine receptors may perturbate circadian timing and consequently behavior during different phases which may explain the enhanced social behavior during the dark phase of the home cage. However, further experiments are required to test this hypothesis.

The current study has some limitations. First, behavioral changes observed in the startle response, open field test, and home cage social interaction test showed nominal significance. Those differences did not reach study-wide significance; therefore, these behavioral assays need to be replicated before application of the findings. Second, further studies are required to decipher the molecular mechanisms underlying the behavioral phenotypes observed in *Glra4* mutant mice. Finally, the study did not address why *GLRA4*, which is considered a pseudogene [11], is reported to be associated with some diseases in humans [17, 18]. *GLRA4* may not function as a protein but may express long non-coding RNA, or the truncated protein itself acts as a dominant-negative. The answer to this question, however, may require the generation of humanized animals harboring *GLRA4*.

In the present study, we showed that the α4 subunit of GlyR is enriched in the brainstem with gradually increasing expression during development. Moreover, *Glra4* mutant mice manifested several behavioral phenotypes such as increased anxiety-like behavior, decreased and delayed acoustic startle response, and enhanced social interaction in the home cage. These findings suggest that glycinergic transmission may modulate startle and social behavior through the α4 subunit of GlyR.

## Supplementary Information

**Additional file 1: Table S1.** List of primer sequences for genes used in RT-qPCR

**Additional file 2:Table S2.** Statistical analysis of behavioral data in Glra4 mutant mice

**Additional file 3**. Additional figures (**Fig. S1**–**S4**). **Fig. S1**: Physical characteristics, muscular strength, motor function, and nociception in Glra4^-/Y^ mice. **Fig. S2**: Activity level of Glra4^-/Y^ mice in the three-chambered social approach test. **Fig. S3**: Depression-related behavior in Glra4^-/Y^ mice. **Fig. S4**: Fear, spatial, and working memory in Glra4^-/Y^ mice.

## Data Availability

The raw data of the behavioral tests and the information about each mouse are accessible on the public database “Mouse Phenotype Database” (http://www.mouse-phenotype.org/). The datasets used and/or analyzed during the current study are available from the corresponding author on reasonable request.

## Abbreviations

GlyRs: Glycine receptors
GlyR α4: Glycine receptor alpha 4
*Glra4 * mutant: Glycine receptor alpha 4 mutant
*GLRA4*: Human glycine receptor alpha 4 subunit
RT-qPCR: Quantitative reverse transcription-polymerase chain reaction
*Plp*1: Proteolipid protein1
*Mbp*: Myelin basic protein
WT: Wild-type
PMD: Pelizaeus Merzbacher disease
CS: Conditioned stimulus
US: Unconditioned stimulus
ANOVA: Analysis of variance
TM: Transmembrane domain
