# Supplementary material for: Comprehensive behavioral analyses of mice with a glycine receptor alpha 4 deficiency
Source: Mol Brain. 2023 May 22;16:44. doi: 10.1186/s13041-023-01033-x (PMC10201759; doi:10.1186/s13041-023-01033-x)
Supplement: Supplementary file 3 — Additional file 3. Additional figures (Fig. S1–S4). Fig. S1: Physical characteristics, muscular strength, motor function, and nociception in Glra4-/Y mice. Fig. S2: Activity level of Glra4-/Y mice in the three-chambered social approach test. Fig. S3: Depression-related behavior in Glra4-/Y mice. Fig. S4: Fear, spatial, and working memory in Glra4-/Y mice. [file 13041_2023_1033_MOESM3_ESM.pdf]

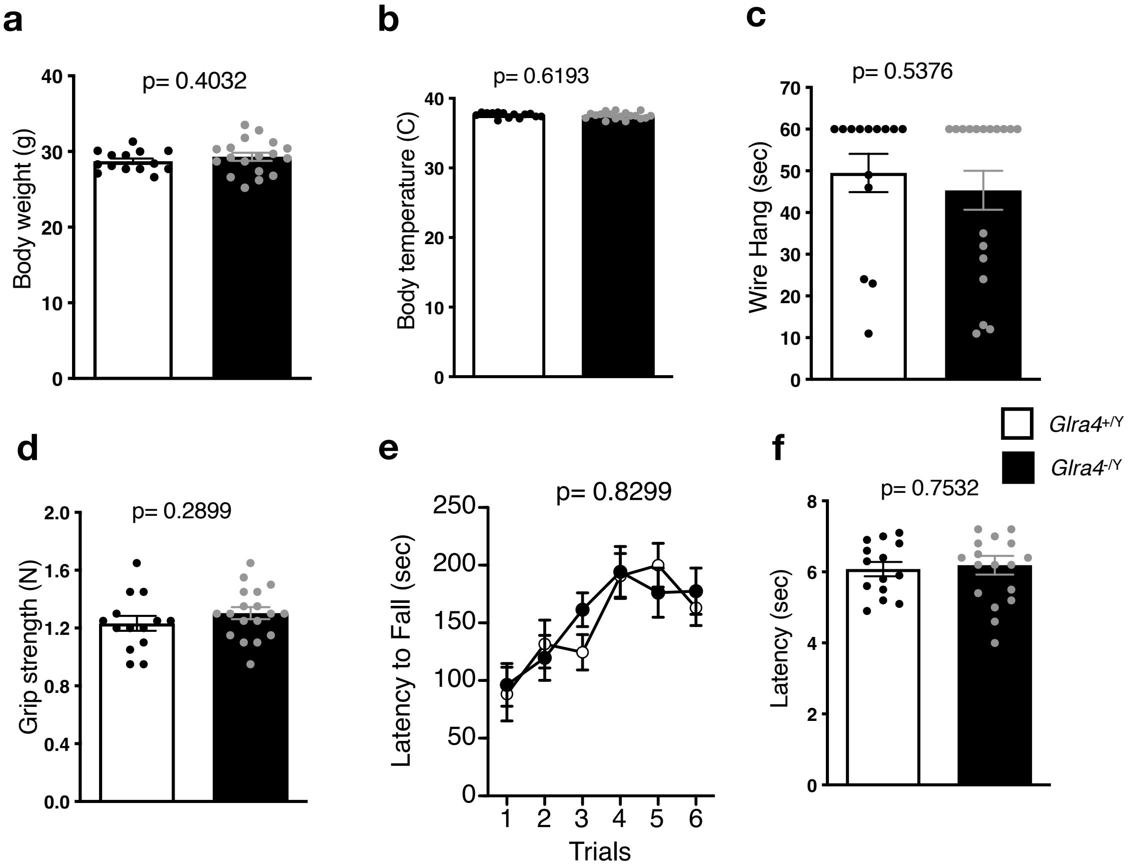

Fig. S1: Physical characteristics, muscular strength, motor function, and nociception in *Glra4*$^{-/Y}$ mice. a) Body weight. b) Body temperature. c) Latency to fall in the wire hang test. d) Grip strength. e) Latency to fall from the rod in the rotarod test. f) Latency to withdraw a paw from a hot plate. n=14 *Glra4*$^{+/Y}$, n=18 *Glra4*$^{-/Y}$. Data are presented as mean ± SEM. The p values indicate the genotype effects in one-way ANOVA (a-d, f), and the genotype effect in two-way repeated-measures ANOVA (e).

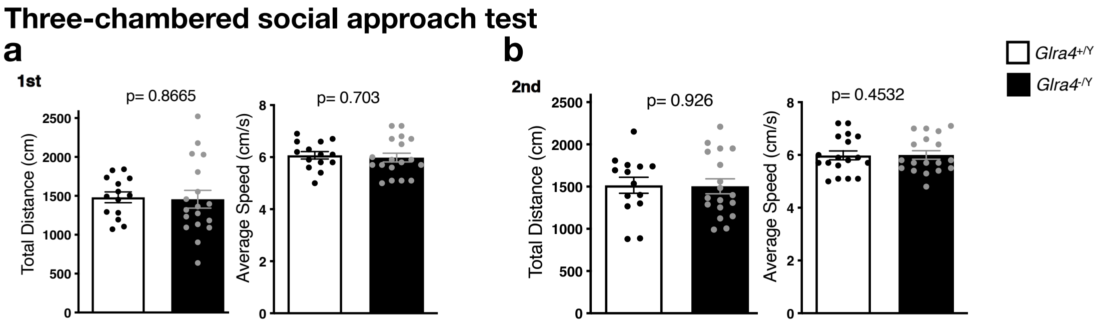

Fig. S2: Activity level of *Glra4*$^{-/Y}$ mice in the three-chambered social approach test. (a–b) Total distance traveled in the first and second sessions in the three-chambered social approach test. n=14 *Glra4*$^{+/Y}$, n=18 *Glra4*$^{-/Y}$. Data are presented as mean±SEM. The p values indicate the genotype effects in one-way ANOVA.

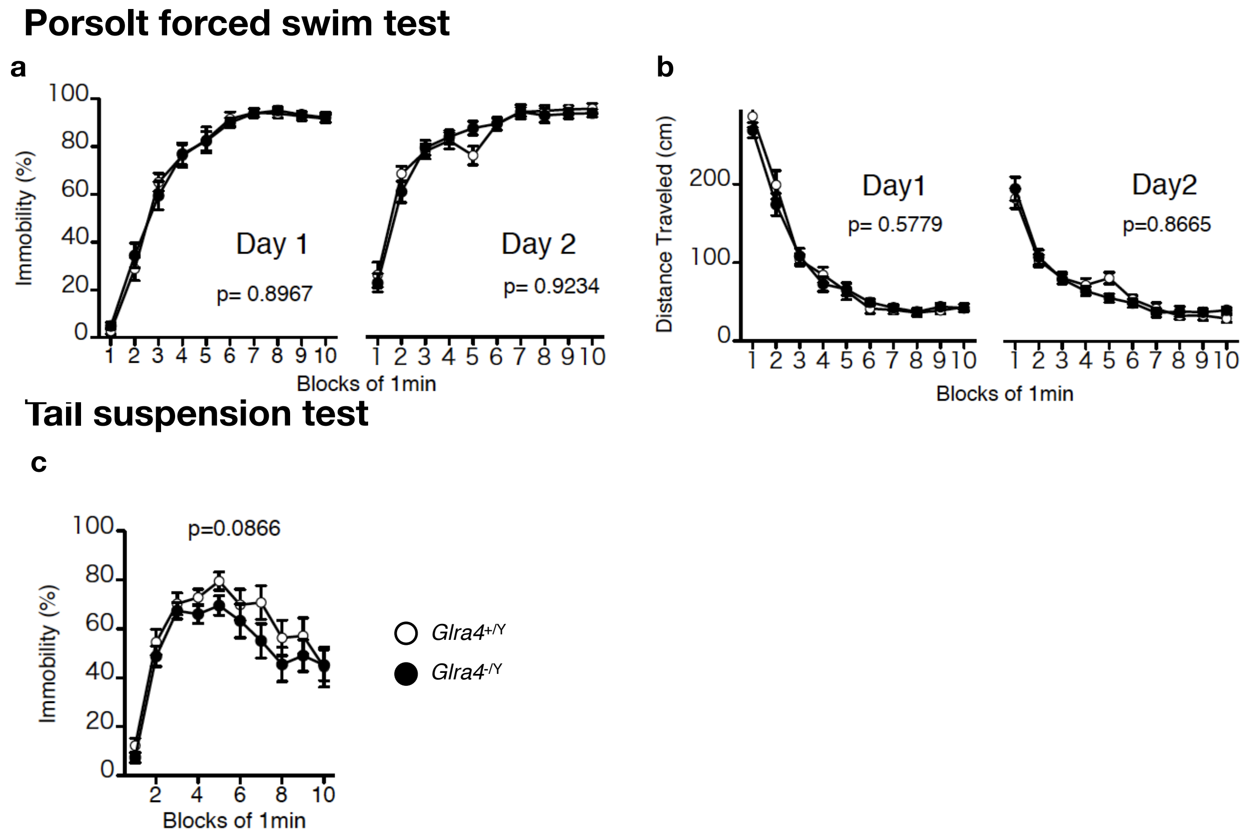

Fig. S3: Depression-related behavior in *Glra4*$^{-/Y}$ mice.

Porsolt forced swim test (a and b): a) Percentage immobility time on day 1 and day 2, and b) distance traveled on day 1 and day 2. c) Percentage immobility time in the tail suspension test. n=14 *Glra4*$^{+/Y}$, n=18 *Glra4*$^{-/Y}$. Data are presented as mean±SEM. The p values indicate the genotype effects in two-way repeated-measures ANOVA.

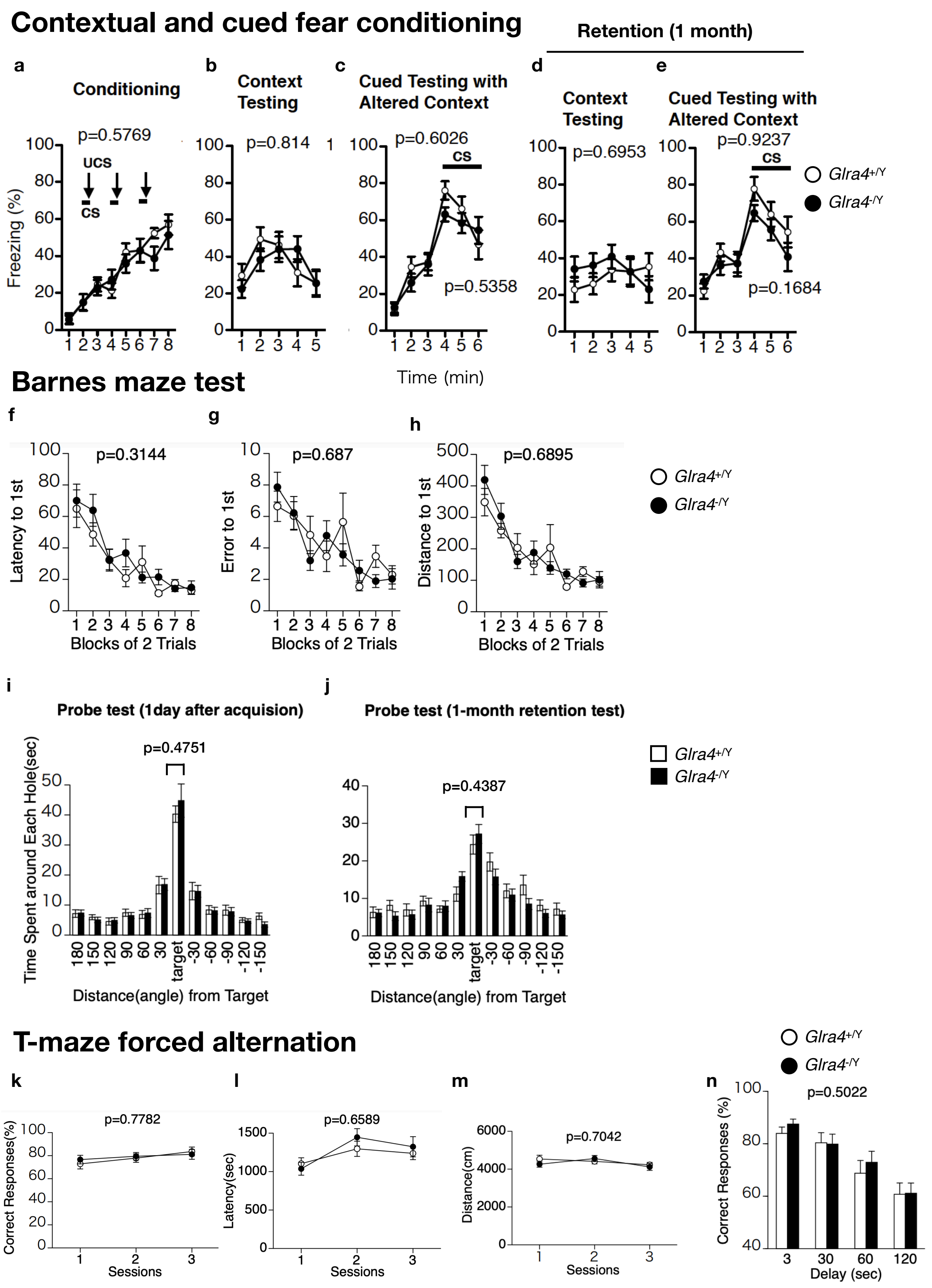
Contextual and cued fear conditioning
Retention (1 month)
a
Conditioning
p=0.5769
UCS
CS
Freezing (%)
b
Context Testing
p=0.814
c
Cued Testing with Altered Context
p=0.6026
CS
p=0.5358
d
Context Testing
p=0.6953
e
Cued Testing with Altered Context
p=0.9237
CS
p=0.1684
Time (min)
Glra4+/Y
Glra4-/Y
Barnes maze test
f
p=0.3144
Latency to 1st
Blocks of 2 Trials
g
p=0.687
Error to 1st
Blocks of 2 Trials
h
p=0.6895
Distance to 1st
Blocks of 2 Trials
Glra4+/Y
Glra4-/Y
i
Probe test (1day after acquision)
p=0.4751
Time Spent around Each Hole(sec)
Distance(angle) from Target
j
Probe test (1-month retention test)
p=0.4387
Distance(angle) from Target
Glra4+/Y
Glra4-/Y
T-maze forced alternation
Glra4+/Y
Glra4-/Y
k
p=0.7782
Correct Responses(%)
Sessions
l
p=0.6589
Latency(sec)
Sessions
m
p=0.7042
Distance(cm)
Sessions
n
p=0.5022
Correct Responses (%)
Delay (sec)

Fig. S4: Fear, spatial, and working memory in *Glra4*$^{-/Y}$ mice. (a-e) Contextual and cued fear conditioning test. Percentage of time freezing in the a) Conditioning trial, b) Context test, c) Cued test, d) Context test after 1-month retention, e) Cued test after 1-month retention. Arrows in the panel indicate shock presentation. US: unconditioned stimulus (shock), CS: conditioned stimulus (tone). (f-j) Barnes maze test. f) Latency to the target hole, g) Number of errors, h) Total distance traveled to the first visit of the correct hole in the initial training period (trials 1–16). Time spent around each hole in probe tests performed at i) 1 day and j) 1 month after the first trial. (k-n) T-maze forced alternation task. k) Percentage of correct responses, l) Latency to finish each session, and m) Total distance traveled. n) Percentage of correct responses in the T-maze delayed forced alternation task. A delay of 3, 30, 60, or 120 s was inserted between the forced-choice run and the free-choice run. n=14 *Glra4*$^{+/Y}$, n=18 *Glra4*$^{-/Y}$. Data are presented as mean ± SEM. The p values indicate the genotype effects in Welch's t-test (i-j), two-way repeated-measures ANOVA (a-h, k-n).
